# Balloon Landmark-Assisted Re-entry Using a Re-entry Catheter for Femoropopliteal Chronic Total Occlusion: A Case Report

**DOI:** 10.7759/cureus.116971

**Published:** 2026-09-26

**Authors:** Tomohide Sato, Eiji Horio, Kazuhisa Kodama, Tomohiro Sakamoto, Junjiroh Koyama

**Affiliations:** 1 Division of Cardiology, Saiseikai Kumamoto Hospital, Kumamoto, JPN

**Keywords:** balloon landmark, chronic kidney disease (ckd), endovascular therapy, evt: endovascular therapy, femoropopliteal chronic total occlusion, re-entry catheter, subintimal angioplasty

## Abstract

Re-entry catheters are effective devices for achieving true lumen re-entry during endovascular therapy (EVT) for femoropopliteal chronic total occlusion (FP-CTO). However, accurate orientation of the catheter may be difficult when angiographic visualization of the target lumen is inadequate. We report a case of successful revascularization using an inflated balloon as a fluoroscopic landmark to facilitate re-entry catheter orientation.

An 81-year-old man presented with symptomatic left lower extremity ischemia caused by FP-CTO. Two previous EVT procedures had failed because guidewire re-entry into the true lumen could not be achieved after subintimal passage. During the third EVT, the retrograde guidewire remained within the true lumen, whereas the antegrade guidewire advanced within the subintimal space. Because angiographic localization of the re-entry point was difficult, a 5 mm balloon was inflated within the subintimal space and used as a fluoroscopic landmark to determine the orientation of the re-entry catheter. After balloon deflation, the re-entry cannula was advanced from the true lumen toward the subintimal space, enabling successful guidewire passage and subsequent stent implantation. The procedure was completed using only 25 mL of contrast medium. The patient's symptoms resolved, and the ankle-brachial index improved from 0.64 to 1.08.

This case suggests that an inflated balloon may serve as a useful fluoroscopic landmark for re-entry catheter orientation when conventional angiographic localization is difficult. In this case, the technique facilitated re-entry catheter orientation and successful guidewire crossing despite limited angiographic visualization. Its potential contribution to contrast minimization, particularly in patients with impaired renal function, requires further evaluation.

## Introduction

Peripheral artery disease (PAD) is a common manifestation of systemic atherosclerosis and is associated with substantial morbidity due to claudication, chronic limb-threatening ischemia, and cardiovascular events. Endovascular therapy (EVT) has become the preferred treatment strategy for many femoropopliteal lesions because of advances in endovascular devices and techniques. However, guidewire crossing remains one of the most technically challenging steps during EVT for femoropopliteal chronic total occlusion (FP-CTO), particularly in long or heavily calcified lesions [1,2].

Re-entry catheters have been developed to facilitate true lumen re-entry after subintimal guidewire passage and have improved the technical success of EVT for FP-CTO. Previous studies have demonstrated that re-entry catheters can shorten procedural and fluoroscopic times and reduce contrast medium use compared with bidirectional approaches [3]. Re-entry devices can also facilitate true lumen re-entry when conventional catheter and guidewire techniques fail to achieve it [4]. Nevertheless, successful use of these devices requires accurate orientation of the re-entry cannula toward the intended target. When the target lumen or intended crossing site is poorly visualized angiographically, determining the spatial relationship between the re-entry catheter and the target may become difficult, thereby complicating precise catheter orientation.

Herein, we report a case of FP-CTO in which conventional angiographic localization was insufficient because the subintimal space could not be adequately visualized. In this technique, an inflated balloon placed within the subintimal space provided a directly visible fluoroscopic landmark for determining the orientation of the re-entry catheter. Unlike previously described target balloon-assisted techniques, the balloon was used as an orientation landmark and was deflated before advancement of the re-entry cannula rather than being intentionally punctured as described in previously reported target balloon-assisted techniques [5,6]. By using an inflated balloon as a fluoroscopic landmark, successful orientation of a re-entry catheter and subsequent revascularization were achieved. This technique may represent a simple and practical option for facilitating guidewire crossing in selected complex FP-CTO cases, particularly when angiographic visualization is limited or minimizing contrast medium use is desirable.

## Case presentation

Patient characteristics and previous endovascular procedures

An 81-year-old man with a history of severe aortic stenosis treated with transcatheter aortic valve replacement, chronic heart failure, chronic kidney disease (CKD), chronic obstructive pulmonary disease, hyperuricemia, and lower extremity PAD presented with progressive intermittent claudication of the left lower extremity, classified as Rutherford category two (moderate claudication) [7]. He had a smoking history of 20 cigarettes per day for 60 years. His regular medications included clopidogrel 75 mg/day, imidapril 2.5 mg/day, bisoprolol fumarate 1.25 mg/day, amlodipine 5 mg/day, spironolactone 25 mg/day, rosuvastatin 5 mg/day, limaprost alfadex 15 μg/day, and febuxostat 10 mg/day. He was also instructed to do home-based walking exercise; however, intermittent claudication persisted and substantially limited his daily activities. Although he remained able to walk within his home, activities requiring walking outside the home, including shopping and going out, were difficult.

On day one, the patient underwent the first EVT at our institution for a chronic total occlusion extending from immediately distal to the origin of the left superficial femoral artery (SFA) to the distal SFA. Mild calcification was present predominantly in the proximal portion of the lesion. Guidewires with tip loads of 1, 3, 10, and 14 g were sequentially used; however, the guidewires predominantly advanced through the subintimal space rather than the true lumen. Attempts to establish communication with the true lumen using 4 mm and 5 mm cutting balloons and a crossing catheter were unsuccessful. Although complete true lumen re-entry could not be achieved, partial communication between the subintimal space and the true lumen resulted in limited antegrade flow, and the procedure was therefore terminated. Intermittent claudication nevertheless persisted after the procedure. The total volume of iodinated contrast medium used during the first procedure was 50 mL.

A second EVT was performed at our institution on day 57. In addition to the guidewires used during the first procedure, a tapered guidewire was employed, and a 4 mm balloon was used in an attempt to achieve true lumen re-entry. However, the guidewire again remained predominantly within the subintimal space, and successful re-entry into the true lumen could not be achieved. The total volume of iodinated contrast medium used during the second procedure was 60 mL. The patient had advanced CKD, and the amount of iodinated contrast medium used during both the first and second procedures had to be intentionally restricted because of concern regarding further deterioration of renal function. Consequently, extensive angiographic assessment using a conventional volume of contrast medium was not feasible. Considering the prolonged procedure and fluoroscopy times together with the need to avoid additional contrast exposure, the second procedure was terminated without successful revascularization. 

Because intermittent claudication persisted and continued to interfere with daily activities despite the two previous EVT attempts, the patient wished to undergo further revascularization. Surgical bypass was considered as an alternative; however, given his advanced age and multiple comorbidities, including CKD, chronic heart failure, previous transcatheter aortic valve replacement, and chronic obstructive pulmonary disease, as well as his preference for an endovascular approach, a third EVT was selected. A dedicated re-entry catheter, which had not been used during the previous procedures, was planned as an alternative strategy to achieve true lumen re-entry. Before the third procedure, the ankle-brachial index (ABI) was 0.84 on the right and 0.64 on the left. Laboratory testing demonstrated advanced renal dysfunction, with a blood urea nitrogen level of 42.9 mg/dL, serum creatinine level of 2.89 mg/dL, and estimated glomerular filtration rate of 17.3 mL/min/1.73 m². The complete laboratory findings are summarized in Table 1. 

**Table 1:** Laboratory findings before the third endovascular procedure

Tests performed	Result	Reference range
White blood cell count (WBC), /μL	6,600	3,300–8,600
Red blood cell count (RBC), ×10⁴/μL	347	386–492
Hemoglobin (Hb), g/dL	10.4	11.6–14.8
Hematocrit (Ht), %	32.5	35.1–44.4
Platelet count (Plt), ×10⁴/μL	22.4	15.8–34.8
Mean corpuscular volume (MCV), fL	93.7	83.6–98.2
Mean corpuscular hemoglobin (MCH), pg	30	27.5–33.2
Mean corpuscular hemoglobin concentration (MCHC), %	32	31.7–35.3
Total protein (TP), g/dL	7.5	6.6–8.1
Albumin (Alb), g/dL	3.9	4.1–5.1
Blood urea nitrogen (BUN), mg/dL	42.9	8–20
Creatinine (Cr), mg/dL	2.89	0.65–1.07
Estimated glomerular filtration rate (eGFR), mL/min/1.73 m²	17.3	≥60
Uric acid (UA), mg/dL	8.4	2.6–7.0
Sodium (Na), mEq/L	138	138–145
Potassium (K), mEq/L	4.08	3.6–4.8
Chloride (Cl), mEq/L	102	101–108
Total bilirubin (T-Bil), mg/dL	0.5	0.4–1.5
Aspartate aminotransferase (AST), U/L	15	13–30
Alanine aminotransferase (ALT), U/L	6	7–23
Lactate dehydrogenase (LDH), U/L	269	124–222
Alkaline phosphatase (ALP), U/L	70	38–113
γ-Glutamyl transpeptidase (γ-GTP), U/L	23	13–64
Total cholesterol (TC), mg/dL	198	142–248
High-density lipoprotein cholesterol (HDL-C), mg/dL	45	38–90
Triglycerides (TG), mg/dL	129	40–234
Low-density lipoprotein cholesterol (LDL-C), mg/dL	110	65–163
Blood glucose, mg/dL	119	73–109
Hemoglobin A1c (HbA1c), %	5.4	4.9–6.0

Lower-extremity duplex ultrasonography performed after the first EVT demonstrated turbulent flow with an increased peak systolic velocity in the midportion of the left SFA, suggesting residual stenosis or partial dissection, with collateral flow distal to the lesion (Figure 1).

**Figure 1:**
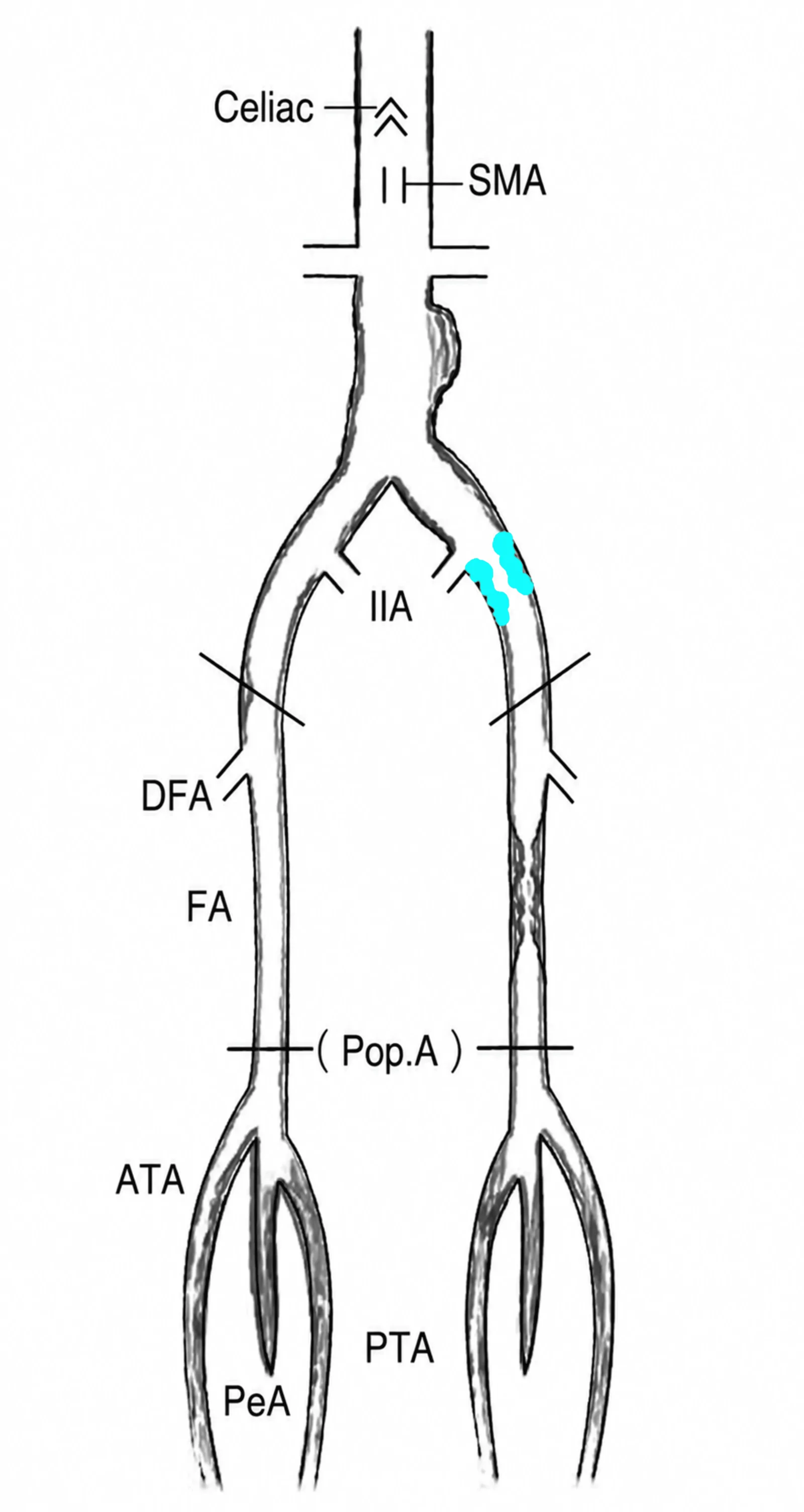
Schematic representation of lower-extremity arterial findings based on ultrasonography Lower-extremity arterial ultrasonography performed after the first endovascular procedure demonstrated turbulent flow with an increased peak systolic velocity of 4.0 m/s in the midportion of the left superficial femoral artery, suggesting residual stenosis or partial dissection. Collateral blood flow was observed distal to the lesion. Occlusion of the left anterior tibial artery and the midportion of the posterior tibial artery was also identified. These ultrasonographic findings are illustrated schematically in the figure. SMA, superior mesenteric artery; IIA, internal iliac artery; DFA, deep femoral artery; FA, femoral artery; Pop.A, popliteal artery; ATA, anterior tibial artery; PTA, posterior tibial artery; PeA, peroneal artery. Image credit: The schematic image was generated using the NEXUS physiological examination system (Fujifilm Medical Co., Ltd., Tokyo, Japan).

Time-of-flight magnetic resonance angiography (TOF-MRA) demonstrated occlusion of the left SFA from its proximal segment, with collateral vessels supplying the distal arterial bed (Figure 2).

**Figure 2:**
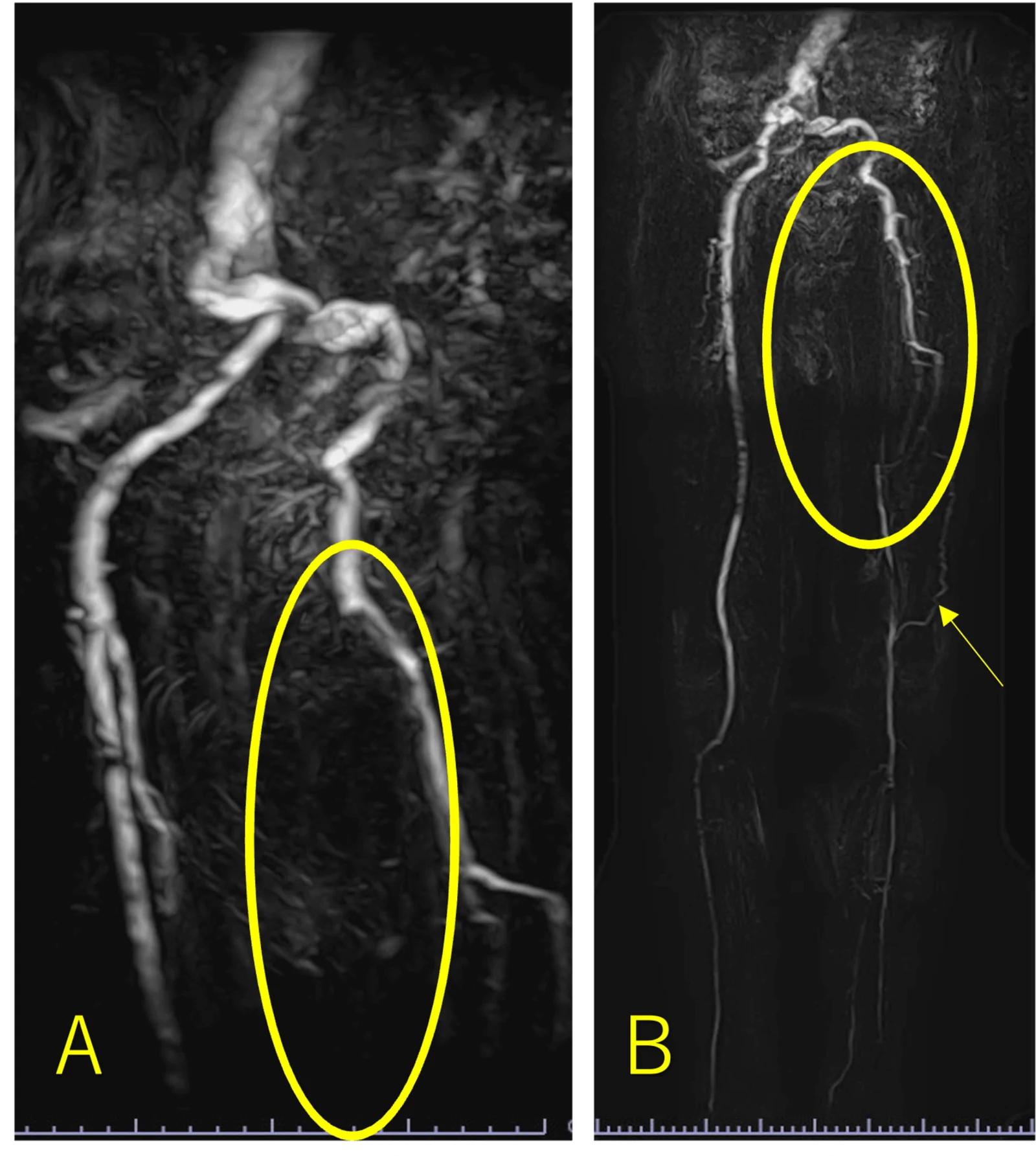
Time-of-flight magnetic resonance angiography of the lower extremities Time-of-flight magnetic resonance angiography (TOF-MRA) demonstrated chronic total occlusion of the left superficial femoral artery from its origin. (A) A magnified view of the left femoral region demonstrates the occluded segment (yellow oval); (B) A whole-leg view demonstrates the long-segment occlusion of the left superficial femoral artery (yellow oval). A collateral vessel from the deep femoral artery to the distal superficial femoral artery is indicated by the yellow arrow.

Endovascular procedure

The third EVT was performed on day 102. Because the two previous procedures had failed to achieve successful wire crossing, a dedicated re-entry catheter was selected as an alternative strategy to achieve revascularization. Given the patient's advanced CKD, minimizing unnecessary contrast administration was also considered important.

A retrograde guidewire was advanced via ultrasound-guided direct puncture of the left popliteal artery, whereas the antegrade guidewire again entered the subintimal space shortly after the origin of the SFA (Figure 3A).

**Figure 3:**
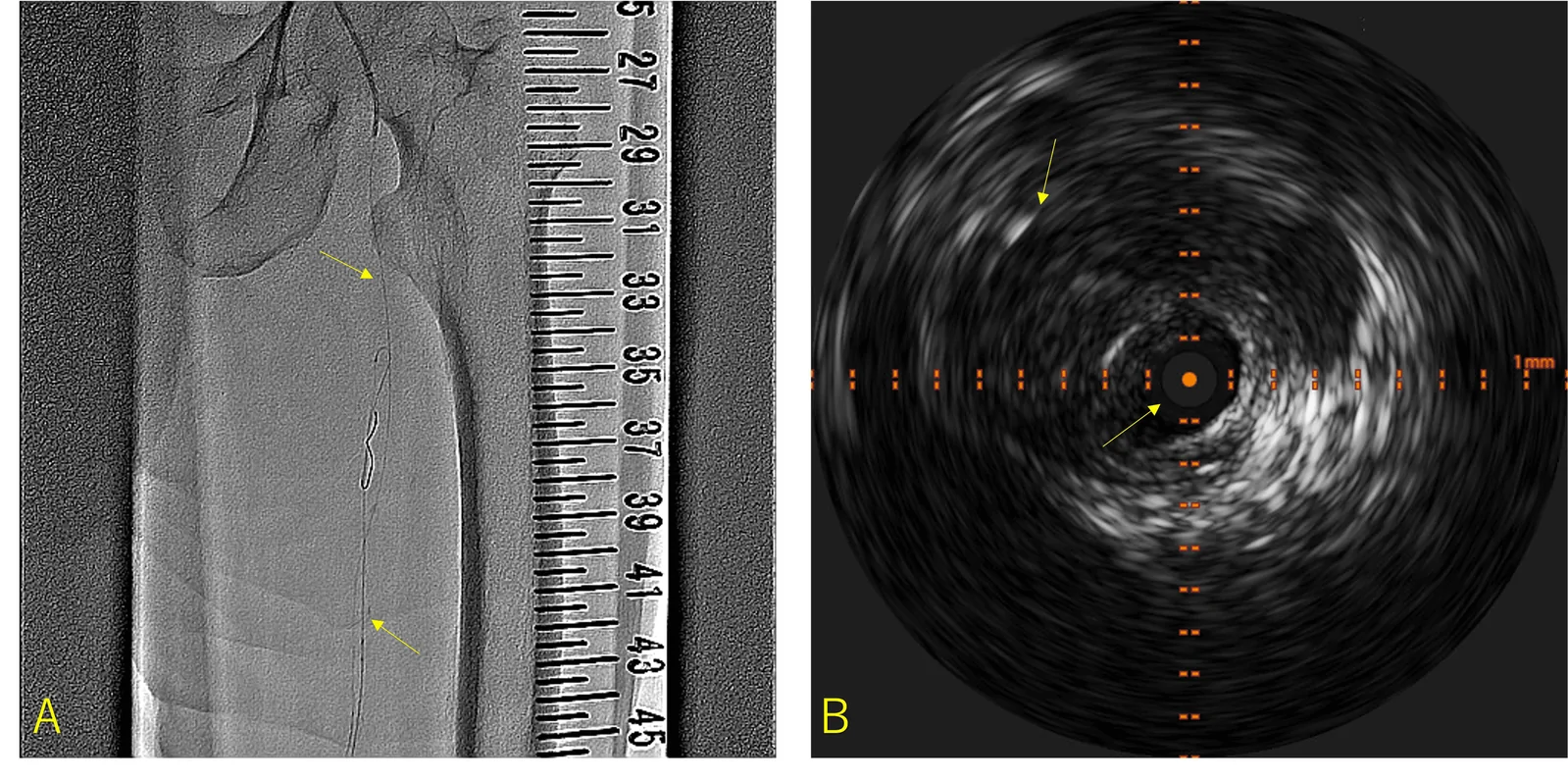
Fluoroscopic and intravascular ultrasound findings demonstrating the guidewire positions (A) Fluoroscopic image showing the positions of the antegrade and retrograde guidewires. The upper arrow indicates the antegrade guidewire within the subintimal space, whereas the lower arrow indicates the retrograde guidewire within the true lumen; (B) Intravascular ultrasound image confirming the guidewire positions. The upper arrow indicates the antegrade guidewire within the subintimal space, whereas the lower arrow indicates the retrograde guidewire within the true lumen.

Intravascular ultrasound (IVUS) confirmed that the retrograde guidewire was located within the true lumen, whereas the antegrade guidewire was positioned within the subintimal space (Figure 3B).

A dedicated re-entry catheter was then advanced retrogradely along the guidewire within the true lumen to the intended re-entry site. 

In conventional use, a re-entry catheter is generally advanced within the subintimal space and directed from the subintimal space toward the true lumen. In the present case, however, we used the device in the opposite direction, advancing it within the true lumen and directing the cannula toward the subintimal space. Angiographic visualization of the antegrade subintimal channel was inadequate, making precise orientation of the re-entry catheter difficult. In addition, repeated contrast injections were undesirable because of the patient's advanced CKD.

Before balloon placement, IVUS was performed from both the antegrade and retrograde approaches to identify the intended crossing site, which was then marked fluoroscopically. After withdrawal of the antegrade IVUS catheter, a 5 mm balloon was advanced over the antegrade guidewire to the fluoroscopically marked site within the subintimal space and inflated, where it served as a fluoroscopic landmark. The retrogradely advanced re-entry catheter was then oriented toward the inflated balloon under fluoroscopic guidance using two orthogonal projections (Right Anterior Oblique or RAO 45° and Left Anterior Oblique or LAO 45°) (Figure 4A). 

**Figure 4:**
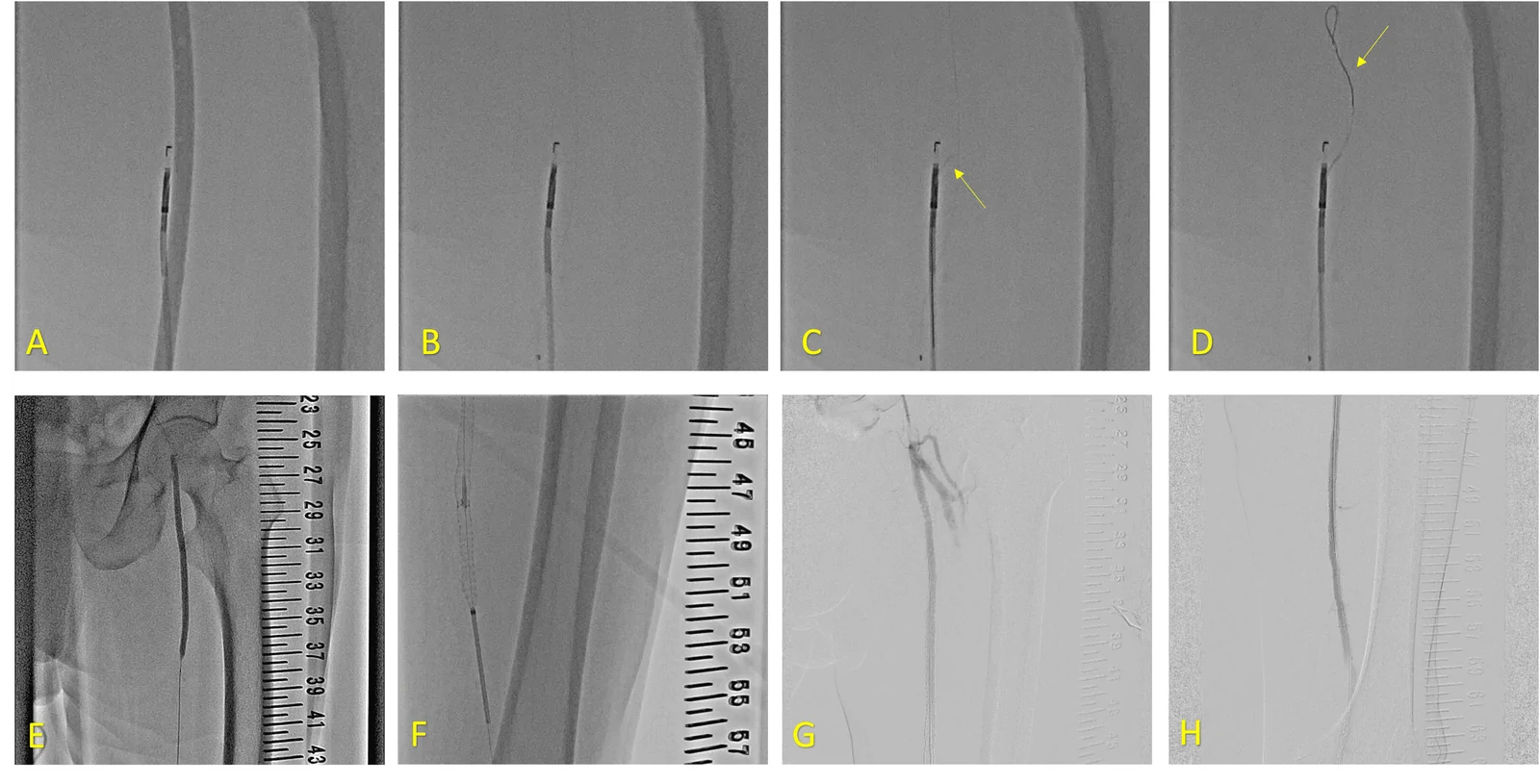
Balloon landmark-assisted re-entry technique and procedural outcome (A) A 5 mm balloon was advanced antegradely into the subintimal space and inflated at the intended crossing site to serve as a fluoroscopic landmark. The re-entry catheter, advanced retrogradely within the true lumen, was oriented toward the inflated balloon; (B) After the appropriate direction of the re-entry catheter had been confirmed, the balloon was deflated before puncture; (C) The cannula of the re-entry catheter was advanced from the true lumen toward the subintimal space (yellow arrow); (D) A guidewire was advanced through the septum into the subintimal space, establishing communication between the true lumen and the antegrade subintimal channel (yellow arrow); (E) Balloon angioplasty was performed using a 5 mm balloon; (F) A 6 mm stent was implanted; (G, H) Final angiography demonstrated satisfactory antegrade flow through the treated femoropopliteal segment.

After the appropriate direction had been confirmed, the balloon was deflated to avoid direct injury to the balloon during puncture (Figure 4B). The cannula of the re-entry catheter was subsequently advanced from the true lumen toward the subintimal space (Figure 4C), and a guidewire was passed through the septum into the subintimal space (Figure 4D). This maneuver successfully established communication between the true lumen and the antegrade subintimal channel.

After successful wire passage, balloon angioplasty was performed using a 5 mm balloon (Figure 4E), followed by implantation of a 6 mm stent (Figure 4F). Final angiography demonstrated satisfactory antegrade flow with three-vessel distal runoff and no significant residual stenosis, flow-limiting dissection, vessel perforation or contrast extravasation, or distal embolization (Figures 4G, 4H). For comparison, the first and second EVT procedures required 288 and 294 min, respectively, with fluoroscopy times of 180 and 130 min, radiation doses of 0.683 and 0.237 Gy, and contrast volumes of 50 and 60 mL, respectively. The third procedure required 202 min, with a fluoroscopy time of 70 min, a radiation dose of 0.133 Gy, and a total iodinated contrast volume of 25 mL.

Clinical outcome

No procedure-related complications occurred. The patient was discharged home after postprocedural examinations revealed no significant abnormalities. The left ABI improved from 0.64 before the procedure to 0.88 on day 103 and further improved to 1.08 on day 145. Intermittent claudication completely resolved after the third EVT and did not recur during follow-up. Serial left ABI values were 1.05 on day 250, 0.92 on day 432, 0.92 on day 502, and 0.97 on day 691, indicating sustained hemodynamic improvement compared with the preprocedural value. Serum creatinine was 2.89 mg/dL before the third EVT and 2.55 mg/dL on day 103, the day after the procedure, with no immediate postprocedural increase. It subsequently decreased to 2.17 mg/dL on day 117. The patient was followed through day 691, approximately 19 months after the third EVT.

## Discussion

This case demonstrates a modified re-entry technique using an inflated balloon as a fluoroscopic landmark in a patient with a chronic total occlusion of the superficial femoral artery after two unsuccessful endovascular procedures. The principal technical features were the orientation of a dedicated re-entry catheter toward the antegrade subintimal channel using an inflated balloon as a fluoroscopic landmark and subsequent puncture from the true lumen into the subintimal space after balloon deflation. This strategy enabled successful revascularization, and the procedure was completed using a total of 25 mL of iodinated contrast medium. Dedicated re-entry catheters are conventionally used after subintimal guidewire passage to facilitate re-entry from the subintimal space into the distal true lumen. The OUTBACK re-entry catheter uses orthogonal fluoroscopic views and T- and L-shaped markers to orient the cannula toward the target lumen [3]. Although these features facilitate directional control, accurate orientation may become challenging when angiographic visualization of the target space is limited. This limitation is particularly relevant in patients in whom repeated contrast injections should be minimized.

The use of an inflated balloon as a target to facilitate re-entry catheter orientation has also been described. Patrone et al. reported target balloon-assisted antegrade or retrograde re-entry in 36 patients, with technical success achieved in 34 patients (94%) [5]. In their technique, the inflated balloon served as a fluoroscopically visible target for the re-entry catheter and was intentionally punctured with the re-entry needle, after which a guidewire was advanced into the balloon lumen to facilitate wire passage and externalization [5]. Similarly, balloon-targeted re-entry using the OUTBACK catheter has been reported in complex peripheral chronic total occlusion interventions [6]. Thus, previous reports have described the use of an inflated balloon as a fluoroscopic target to facilitate re-entry catheter guidance in challenging chronic total occlusion interventions.

Our approach was conceptually similar to these target balloon-assisted techniques but differed in several procedural aspects. In the previously described target balloon-assisted approach, the re-entry cannula was directed toward and intentionally advanced into the inflated target balloon, after which the guidewire was passed into the balloon lumen to facilitate wire passage and externalization [5,6]. In contrast, in the present case, the balloon placed within the antegrade subintimal space was used only as a fluoroscopic landmark for orientation. The re-entry catheter was advanced retrogradely within the true lumen, and its cannula was directed toward the subintimal space. After the appropriate direction had been confirmed, the balloon was deflated before cannula advancement and was not intentionally punctured. The guidewire was then passed through the septum directly from the true lumen into the subintimal space, rather than into the balloon lumen. Thus, our technique can be regarded as a modification of previously reported target balloon-assisted re-entry techniques, in which the balloon was used primarily as a fluoroscopic landmark for catheter orientation while intentional balloon puncture was avoided. This approach may provide an alternative strategy in selected cases in which conventional re-entry is difficult and repeated contrast injections are undesirable.

Minimizing iodinated contrast exposure was particularly important in this patient because of advanced CKD. Kawasaki et al. reported that the use of a re-entry catheter for FP-CTO was associated with lower contrast volume and shorter procedural and fluoroscopic times compared with a bidirectional approach [3]. Furthermore, contrast-sparing approaches have been reported in patients with severe renal impairment. Nojima et al. described successful revascularization of bilateral FP-CTOs using a re-entry catheter under carbon dioxide angiographic guidance in a patient with severe renal impairment [8]. In the present patient, iodinated contrast administration had already been intentionally restricted during the first two procedures because of concern for further deterioration of renal function. During the third procedure, the inflated balloon provided a readily visible fluoroscopic landmark for re-entry catheter orientation, and successful revascularization was achieved using 25 mL of iodinated contrast medium. However, because this is a single case without a direct procedural comparison, the extent to which the balloon landmark-assisted technique contributed to the lower contrast volume cannot be determined. Furthermore, contrast minimization should not be interpreted as evidence of prevention of contrast-associated kidney injury. Further experience is required to determine whether this technique can reproducibly facilitate contrast minimization in patients for whom contrast exposure is a concern.

Several limitations should be acknowledged. First, this technique was demonstrated in a single patient and by a single procedural team; therefore, its safety, feasibility, and reproducibility cannot be established from this case. In particular, its reproducibility across different operators, re-entry devices, and lesion anatomies remains unknown. Second, advancing a re-entry cannula from the true lumen toward the subintimal space represents a nonstandard direction of use and may carry risks such as vessel perforation or unintended tissue injury. Precise understanding of the spatial relationship among the true lumen, subintimal space, balloon, and re-entry catheter is therefore essential. Third, although deflation of the balloon before cannula advancement avoided intentional balloon puncture, the optimal balloon diameter, inflation pressure, and spatial relationship between the balloon and the re-entry catheter remain uncertain. The feasibility of balloon landmark-assisted orientation may also depend on lesion anatomy. Severe calcification or marked vessel tortuosity may limit device advancement and precise catheter orientation, whereas a particularly narrow subintimal space may make balloon placement and inflation difficult. In very long CTOs, maintaining an appropriate spatial relationship between the balloon and the re-entry catheter at the intended crossing site may also be challenging. Further experience is required to determine the anatomical and procedural settings in which this technique may be most appropriate.

## Conclusions

A modified re-entry technique using an inflated balloon as a fluoroscopic landmark enabled successful revascularization of a difficult superficial femoral artery CTO after two previous unsuccessful endovascular procedures. By using the balloon to guide re-entry catheter orientation and deflating it before puncture, communication between the true lumen and subintimal space was successfully established without intentional balloon puncture. In this case, the principal demonstrated benefit of the technique was facilitation of re-entry catheter orientation and successful guidewire crossing. Although the procedure was completed using a limited volume of iodinated contrast medium, the extent to which this technique contributed to contrast minimization remains uncertain. Further evaluation in additional cases and comparative experience is required to establish its safety, reproducibility, optimal technical parameters, and applicability across different FP-CTO anatomies.

## References

[1] Gornik HL, Aronow HD, Goodney PP (2024). 2024 ACC/AHA/AACVPR/APMA/ABC/SCAI/SVM/SVN/SVS/SIR/VESS guideline for the management of lower extremity peripheral artery disease: a report of the American College of Cardiology/American Heart Association joint committee on clinical practice guidelines. Circulation.

[2] Mazzolai L, Teixido-Tura G, Lanzi S (2024). 2024 ESC guidelines for the management of peripheral arterial and aortic diseases. Eur Heart J.

[3] Kawasaki D, Fukunaga M, Nakata T, Kato M, Ohkubo N (2017). Comparison of the OUTBACK(®) elite reentry catheter and the bi-directional approach after failed antegrade approach for femoro-popliteal occlusive disease. J Atheroscler Thromb.

[4] Jacobs DL, Motaganahalli RL, Cox DE, Wittgen CM, Peterson GJ (2006). True lumen re-entry devices facilitate subintimal angioplasty and stenting of total chronic occlusions: initial report. J Vasc Surg.

[5] Patrone L, Theivacumar NS, Dharmarajah B (2023). Target balloon-assisted antegrade and retrograde use of re-entry catheters in complex chronic total occlusions. J Cardiovasc Dev Dis.

[6] Rippel K, Ruhnke H, Jehs B, Decker J, Kroencke T, Scheurig-Muenkler C (2021). Targeted tibio-peroneal re-entry during subintimal revascularization using the Outback® catheter. CVIR Endovasc.

[7] Rutherford RB, Baker JD, Ernst C, Johnston KW, Porter JM, Ahn S, Jones DN (1997). Recommended standards for reports dealing with lower extremity ischemia: revised version. J Vasc Surg.

[8] Nojima Y, Nanto S, Adachi H, Ihara M, Kurimoto T (2017). Combination of carbon dioxide angiography and Outback® elite for revascularization of a patient with renal insufficiency with bilateral femoropopliteal chronic total occlusions. Case Rep Cardiol.

